# 655. Analysis of Microbiome Diversity and Secondary Bile Acid Synthesis Following SER-109 or Placebo in Patients with First Recurrent or Multiply Recurrent *Clostridioides difficile* Infection

**DOI:** 10.1093/ofid/ofad500.718

**Published:** 2023-11-27

**Authors:** Colleen R Kelly, Colleen S Kraft, Tim Straub, Kevin Litcofsky, Jennifer R Wortman, Stuart H Cohen, Charles Berenson, Barbara McGovern, Brooke Hasson, Dina Hot, Darrell Pardi, Christopher Ford, Matthew Henn

**Affiliations:** Warren Alpert Medical School of Brown University, Providence, Rhode Island; Emory University, Atlanta, GA; Seres Therapeutics, Cambridge, MA, Cambridge, Massachusetts; Seres Therapeutics, Inc., Cambridge, MA; Seres Therapeutics, Cambridge, MA, Cambridge, Massachusetts; University of California, Davis, Sacramento, CA; State University of New York at Buffalo, Buffalo, New York; Seres Therapeutics, Inc., Cambridge, MA; Seres Therapeutics, Cambridge, MA, Cambridge, Massachusetts; Aimmune Therapeutics, a Nestlé Health Science company, Brisbane, CA, Brisbane, California; Mayo Clinic, Rochester, MN; Seres Therapeutics, Inc, Cambridge, MA; Seres Therapeutics, Cambridge, MA, Cambridge, Massachusetts

## Abstract

**Background:**

Treatment for first recurrent *Clostridioides difficile* infection (frCDI) or multiply recurrent CDI (mrCDI) is complex and may include antibiotics, monoclonal antibodies, or fecal transplantation. Improved understanding of rCDI pathogenesis can help guide treatment. We report microbiome findings of patients (pts) with frCDI and mrCDI from a post hoc analysis of two phase 3 trials with fecal microbiota spores, live-brpk (FMS; formerly SER-109), an FDA-approved, microbiota-based therapeutic comprised of Firmicutes spores.

**Methods:**

Stool samples were collected from pts with mrCDI in ECOSPOR III (n=158) and with frCDI or mrCDI in ECOSPOR IV (n=218) at baseline (pre-dose) and Week 1 post treatment. Shannon diversity was calculated from species profiles from analysis of whole metagenomic sequencing data using MetaPhlAn2. Primary (1°BA) and secondary bile acid (2°BA) concentrations were measured via targeted liquid chromatography–mass spectrometry panel. Subgroup differences were analyzed with linear mixed models.

**Results:**

At baseline, Shannon diversity was not significantly different between mrCDI and frCDI groups (*P*>0.05). At Week 1, Shannon diversity (**Fig 1**) was greater vs baseline (*P*< 0.001); gains were not significantly different between subgroups (*P*>0.05). For pts receiving FMS vs placebo in ECOSPOR III, greater reductions in 1°BA and increases in 2°BA from baseline–Week 1 were observed, similar to FMS-treated pts in ECOSPOR IV. At Week 1, 2°BA concentrations were greater vs baseline (*P*< 0.001); similar gains were seen between subgroups (*P*>0.05) (**Fig 2**). Consistent with these microbiome findings, in an integrated analysis, rCDI rates with FMS at Week 8 were low in both subgroups (frCDI, 6.5% [5/77] vs mrCDI, 10.3% [28/271]).

**Figure:**
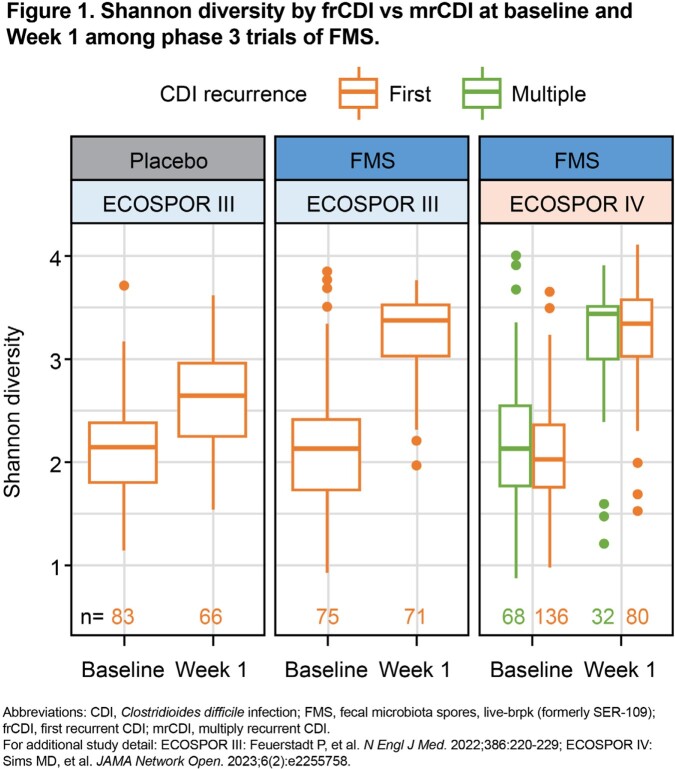

**Figure:**
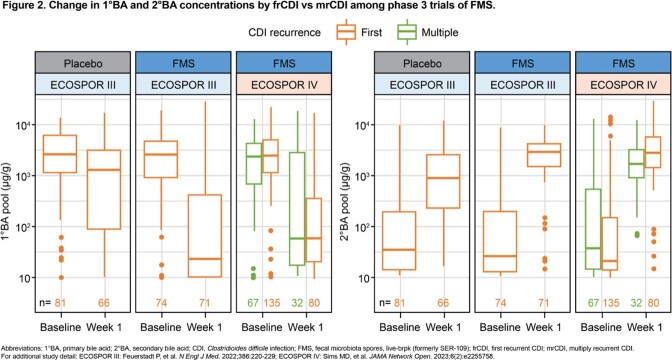

**Conclusion:**

After antibiotics, pts with frCDI or mrCDI had low Shannon diversity and 2°BA concentrations that were no different between subgroups, highlighting a need for microbiome restoration. Both subgroups showed rapid, significant improvement in Shannon diversity and 2°BA concentrations and reductions in 1°BA after FMS, with comparable clinical outcomes. These data suggest that regardless of number of prior CDI episodes, FMS therapy following antibiotics may be an optimal treatment.

**Disclosures:**

**Colleen R. Kelly, MD**, Openbiome: Advisor/Consultant|Sebela Pharmaceuticald: Advisor/Consultant **Tim Straub, MS**, Seres Therapeutics: Employee|Seres Therapeutics: Stocks/Bonds **Kevin Litcofsky, PhD**, Seres Therapeutics: inventor on patents assigned to Seres Therapeutics|Seres Therapeutics: Employment|Seres Therapeutics: Stocks/Bonds **Jennifer R. Wortman, MS**, Seres Therapeutics: Employee|Seres Therapeutics: Stocks/Bonds **Barbara McGovern, MD**, Seres Therapeutics: Stocks/Bonds **Brooke Hasson, PhD**, Sage Therapeutics: Stocks/Bonds|Seres Therapeutics: Stocks/Bonds **Dina Hot, PhD**, Aimmune Therapeutics: Employee **Darrell Pardi, MD**, Abbvie: Advisor/Consultant|AMT: Grant/Research Support|BI: Advisor/Consultant|Immunic: Advisor/Consultant|Rise: Advisor/Consultant|Seres Therapeutics: Advisor/Consultant|Seres Therapeutics: Grant/Research Support|Summit: Advisor/Consultant|Takeda: Grant/Research Support|Vedanta: Advisor/Consultant|Vedanta: Grant/Research Support **Christopher Ford, PhD**, Seres Therapeutics: Employee|Seres Therapeutics: Stocks/Bonds **Matthew Henn, PhD**, Seres Therapeutics: Employee|Seres Therapeutics: Stocks/Bonds

